# Impact of Emergency Department Crowding on Delays in Acute Stroke Care

**DOI:** 10.5811/westjem.2020.5.45873

**Published:** 2020-07-08

**Authors:** Todd A. Jaffe, Joshua N. Goldstein, Brian J. Yun, Mark Etherton, Thabele Leslie-Mazwi, Lee H. Schwamm, Kori S. Zachrison

**Affiliations:** * Harvard Affiliated Emergency Medicine Residency at Massachusetts General Hospital and Brigham and Women’s Hospital, Department of Emergency Medicine, Boston, Massachusetts; † Massachusetts General Hospital, Department of Emergency Medicine, Boston, Massachusetts; ‡ Massachusetts General Hospital, Department of Neurology, Boston, Massachusetts

## Abstract

**Introduction:**

Delays in identification and treatment of acute stroke contribute to significant morbidity and mortality. Multiple clinical factors have been associated with delays in acute stroke care. We aimed to determine the relationship between emergency department (ED) crowding and the delivery of timely emergency stroke care.

**Methods:**

We used prospectively collected data from our institutional Get with the Guidelines-Stroke registry to identify consecutive acute ischemic stroke patients presenting to our urban academic ED from July 2016–August 2018. We used capacity logs to determine the degree of ED crowding at the time of patients’ presentation and classified them as ordinal variables (normal, high, and severe capacity constraints). Outcomes of interest were door-to-imaging time (DIT) among patients potentially eligible for alteplase or endovascular therapy on presentation, door-to-needle time (DTN) for alteplase delivery, and door-to-groin puncture (DTP) times for endovascular therapy. Bivariate comparisons were made using t-tests, chi-square, and Wilcoxon rank-sum tests as appropriate. We used regression models to examine the relationship after accounting for patient demographics, transfer status, arrival mode, and initial stroke severity by the National Institutes of Health Stroke Scale.

**Results:**

Of the 1379 patients with ischemic stroke presenting during the study period, 1081 (78%) presented at times of normal capacity, 203 (15%) during high ED crowding, and 94 (7%) during severe crowding. Median DIT was 26 minutes (interquartile range [IQR] 17–52); DTN time was 43 minutes (IQR 31–59); and median DTP was 58.5 minutes (IQR 56.5–100). Treatment times were not significantly different during periods of higher ED utilization in bivariate or in multivariable testing.

**Conclusion:**

In our single institution analysis, we found no significant delays in stroke care delivery associated with increased ED crowding. This finding suggests that robust processes of care may enable continued high-quality acute care delivery, even during times with an increased capacity burden.

## INTRODUCTION

Delays in timely identification, imaging, and treatment of acute stroke are associated with significant morbidity and mortality.^1^,^2^ To ensure timely delivery of care, hospitals develop robust processes to promptly identify and treat patients presenting with concern for acute stroke.^3^–^7^ National guidelines recommend administration of alteplase within 60 minutes of patient presentation, and achieving this target is dependent on timely imaging and appropriate utilization of scarce emergency department (ED) resources.^8^,^9^ The availability of many critical resources may be further threatened with the increasing prevalence of ED crowding.^10^–^12^ Previous studies have demonstrated the association of ED crowding with patient safety concerns, delays in care, and even patient mortality.^13^–^15^

Data regarding the relationship between ED crowding and acute stroke care in particular are limited. One study found that among patients presenting with acute symptoms (within three hours), imaging and thrombolysis times were not affected by ED crowding,^16^ whereas another reported that increased crowding was associated with poorer performance on door-to-imaging times (DIT).^17^

Given conflicting findings, we sought to investigate the relationship between ED crowding and timely imaging and treatment of acute stroke in our high-volume, urban, academic ED. We hypothesized that increased crowding would be associated with delays in imaging, alteplase delivery, and time-to-groin puncture for patients undergoing endovascular therapy. We further hypothesized that other factors associated with stroke care, such as higher stroke severity, may mitigate these delays during times of increased crowding.

## METHODS

### Data Source, Study Setting, and Population

This was a retrospective analysis of prospectively collected data on consecutive ischemic stroke patients presenting to a single, urban, academic comprehensive stroke center hospital with over 108,714 ED visits in 2017. The ED resources for acute stroke care include two dedicated ED computed tomography (CT) scanners as well as an in-person neurology team (24/7 availability). The CT scanners are located adjacent to the ED with a <2-minute stretcher transport from high acuity rooms. Code stroke is activated by ED care team members when a patient presents with signs or symptoms concerning for acute stroke. Code stroke activation results in a group page sent immediately to the ED neurology team, ED pharmacist, ED radiology team, and CT technologist. The CT scanner is then cleared and held for evaluation of the patient.

We used data from the institutional Get with the Guidelines-Stroke dataset, which includes patient demographics, and detailed clinical data including time of presentation, time of imaging, stroke severity (measured by National Institutes of Health Stroke Scale [NIHSS] score), time of alteplase administration, and time of puncture for endovascular therapy.^18^,^19^ We included all patients over 18 years of age with a final diagnosis of ischemic stroke, who presented through the ED between July 2016–August 2018.

We matched patients’ time of presentation in the stroke registry data with data from our ED capacity logs. The log documents the state of ED utilization at all times on a three-point ordinal scale of normal capacity, high capacity, or severe capacity constraints. High capacity-constraint status is automatically triggered when all monitored bays and half of the monitored hallway stretchers are occupied. A severe capacity-constraint status is triggered when all monitored beds, including bays and stretchers, are occupied. Patients in our registry were cross-referenced against ED capacity logs to determine the capacity state at time of arrival for each patient.

**Population Health Research Capsule**

What do we already know about this issue?

*Delays in care of acute stroke lead to morbidity and mortality. ED crowding has also been associated with delays for other disease processes*.

What was the research question?

For patients presenting with acute stroke, is ED crowding associated with delays in care?

What was the major finding of the study?

*ED crowding was not associated in delays in care for patients presenting with acute stroke*.

How does this improve population health?

*Our findings suggest that robust systems may be protective in times of increased capacity burden. Further study may help identify other disease processes to target*.

**Figure 1:**
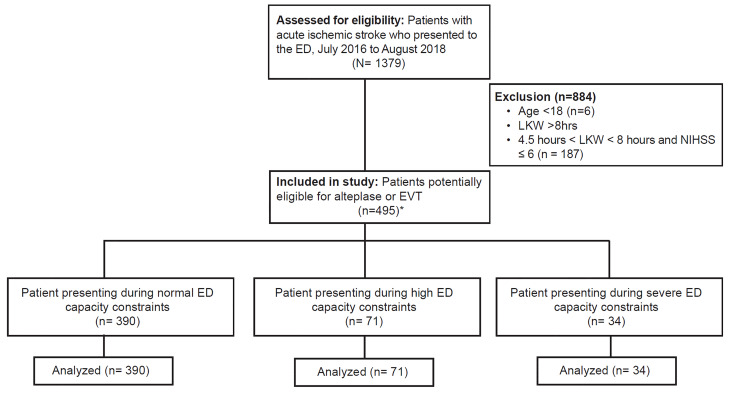
CONSORT diagram for patient inclusion criteria by study outcome. *DIT analysis includes 298/495 patients who were non-transfers. *ED*, emergency department; *LKW*, last known well; *NIHSS*, National Institutes of Health Stroke Scale; *EVT*, endovascular therapy; *DIT*, door-to-imaging time.

Because our objective was to determine whether time-dependent metrics were influenced by ED capacity constraints, we focused this analysis on patients with acute stroke who were potentially eligible for intervention on presentation (Figure 1). This included patients potentially eligible for alteplase (presenting within 4.5 hours of last known well [LKW] for all patients regardless of illness severity), and those potentially eligible for endovascular therapy (presenting within eight hours of LKW with moderate or severe disability, defined as NIHSS ≥ 6).

### Outcomes of Interest

Outcomes of interest were DIT, door-to-needle (DTN) time for alteplase delivery, and door-to-groin puncture time (DTP) for endovascular therapy. For the DIT analysis, we excluded transferred patients to focus on patients in whom previous imaging had not yet been completed. We secondarily examined DIT among all alteplase-treated patients. The DTN analysis included all patient arriving within 4.5 hours of LKW time and treated with alteplase. The DTP analysis included all patients arriving within eight hours of LKW time with NIHSS ≥6 who received endovascular therapy. In addition to our primary outcomes of interest (DIT, DTN, and DTP) we also examined compliance with guideline-recommended dysphagia screening and 25- and 60-minute windows for DIT and DTN, respectively.

### Statistical Analysis

Our independent variable of interest was ED crowding at the time of patient presentation, as defined above. We used t-tests, chi-square, and Wilcoxon rank-sum tests as appropriate for bivariate comparisons. We used regression models to examine the relationship between ED crowding and outcomes of interest after accounting for patient age, gender, transfer status, arrival mode, and stroke severity (based on NIHSS). The covariates listed above included in the model were determined a priori based on clinical experience and prior literature.^1^–^5^,^20^–^23^ We conducted analyses using Stata 14.2 (StataCorp, College Station, Texas). The Massachusetts General Hospital Institutional Review Board approved the study and did not require informed consent for this retrospective data analysis.

## RESULTS

We identified 1379 ED patients with ischemic stroke during the study period. Of this population, 495 were potentially eligible for alteplase or endovascular therapy on presentation. Patient characteristics for this cohort are included in Table 1. Seventy-nine percent of patients with acute ischemic stroke presented in times of normal utilization, while 14% presented during high crowding and 7% presented during times of severe crowding. Patients were more likely to present as a transfer to our ED during times of normal capacity, and patients presenting during severe crowding had lower stroke severity than patients presenting during normal and high-capacity constraints; there were otherwise no other patient-level differences associated with differences in ED capacity status (Table 1).

We further assessed how the distribution of increased crowding for stroke patients compared with the general population. During the study period, our ED had normal capacity constraints 78% of the time, with increased and severe crowding 12% and 10% of the time, respectively. Eighty-one percent of increased crowding activations occurred Monday-Thursday, with the median time of activation 1:35 pm.

### Door-to-Imaging Times

**Figure 2:**
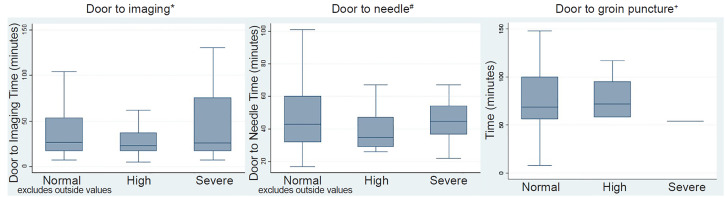
Study outcomes by capacity constraints on the effect of timely treatment of stroke patients. *Includes 286 patients with complete data who were non-transfers, and potentially eligible for alteplase or endovascular therapy. ^#^Includes 82 patients who were not transferred and were treated with alteplase within 4.5 hours of presentation. ^+^Includes all 52 patients who were eligible and received endovascular therapy.

Of the 1379 patients in our sample, 298 patients presented directly (non-transfers) and were potentially eligible for alteplase or endovascular therapy (presented within 4.5 hours of LKW with any stroke severity or within eight hours of LKW with NIHSS score of 6 or greater). Median DIT among this cohort was 26 minutes (interquartile range [IQR] 17–52) and did not significantly differ by ED capacity constraints at time of presentation in bivariate testing (Table 2 and Figure 2), or in multivariable regression after accounting for patient characteristics, EMS arrival, and stroke severity (Supplementary Table). EMS arrival was independently associated with faster DIT. Median DIT among the 82 alteplase-treated patients was 18 minutes (IQR 14–26) and did not significantly differ by ED crowding at time of presentation in bivariate testing (Table 2).

### Door-to-Needle Time for Alteplase Receipt

Among the 82 alteplase-treated patients in our sample, median DTN was 43 minutes (IQR 31–59) and did not significantly vary by ED capacity status at time of presentation in bivariate testing (Table 2, Figure 2) or after accounting for patient characteristics, stroke severity, and EMS arrival (Supplementary Table). Of these patients, 78% had DTN within 60 minutes of arrival.

### Door-to-Groin Puncture for Endovascular Therapy

Among the 52 patients who received endovascular therapy, median DTP was 68.5 minutes (IQR 56.5–100 minutes), and DTP times did not vary by ED capacity status at time of presentation in bivariate testing (Table 2, Figure 2), or after accounting for patient characteristics, stroke severity, and EMS arrival (Supplementary Table).

## DISCUSSION

This study investigated the relationship between ED crowding and prompt recognition and management of patients with acute ischemic stroke. We found no significant difference in time to imaging, administration of alteplase, or to endovascular therapy when the ED was experiencing high or severe capacity constraints. This suggests that robust, protocolized systems in place to address the time-sensitive requirements of stroke treatment may be protective against increasing ED capacity constraints.

Previous data regarding the impact of ED crowding on stroke evaluation and treatment is limited and mixed. Chatterjee et al found that ED crowding was not associated with delays in imaging for patients presenting less than three hours from symptoms onset. However, the study did demonstrate delays if symptoms had been present for longer, suggesting that less emergent care may be delayed in times of worsening ED crowding.^16^ Recently, a study by Reznek et al found an association between ED crowding and failure to comply with DIT goals under 25 minutes.^17^ This is in contrast to our results, in which crowding was not associated with delays in DIT.

There are multiple potential explanations for this difference. First, our institution has multiple CT scanners available to the ED, which may be protective in times of increased volume. Additionally, differences in the populations included in the studies may have contributed to the difference in results. The population included in the Reznek et al study included all patients in whom a “code stroke” was activated, with symptom onset within 12 hours. This may have led to the inclusion of patients who were not candidates for acute treatment, and as such the time-dependency of their imaging may have been considered less critical. The patients included in our study were those with a retrospective diagnosis of acute stroke and who were also potentially eligible for treatment on presentation. Given that the patients included in our sample had potential for intervention on arrival to the ED, expediting their evaluation may have been even further prioritized. Thus, these patients experienced no delays in care in times of increased crowding.

Other studies have also examined other patient or clinical factors in addition to ED crowding that are associated with prompt imaging and management of acute stroke. These factors have included gender, symptom severity, and mode of ED arrival.^21^–^23^ We did not find any disparities by gender or race/ethnicity; however, consistent with previous reports, we did find an association between EMS arrival and faster DIT.

Our study expands on previous work by assessing the relationship between timely stroke care and capacity constraints, and adds a novel analysis of DTP for endovascular therapy. Our findings underscore the value of dedicated ED protocols and processes to ensure high-quality delivery of time-critical care irrespective of ED volume. Having a dedicated ED stroke team, ED pharmacist, and neuroradiology support in the ED may reduce any variation in imaging times that capacity constraints would otherwise impose. However, the availability of these resources may be both institution- and disease-specific. Some institutions with resource constraints may be more likely to experience delays in acute care with only marginal increases in ED crowding. Further study may identify what level of crowding may lead to delays for stroke care as well as the resources needed to protect against capacity constraints.

Our results have potential implications for the organization of stroke systems of care. In the prehospital triage of patients with suspected stroke due to large vessel occlusion (LVO), it is hypothesized that transport directly to thrombectomy-capable centers could introduce harm due to possible over-triage. There is concern that this action may lead to increased crowding and worse outcomes for patients at these hospitals. However, our results suggest that for patients presenting within the treatment windows for alteplase or thrombectomy, crowding does not contribute to slower treatment times. Thus, protocoled care may enable treatment without delays, regardless of crowding conditions, in scenarios of transport directly to a thrombectomy-capable hospital.

As crowding and capacity challenges continue to be a pervasive issue for many EDs, developing and maintaining efficient processes to ensure high-quality care for high acuity, time-critical patients is paramount. Studies have highlighted how defined systems of care for critical disease processes can protect against delays in care, yet even these results have been mixed. For example, there are conflicting results regarding the impact of ED crowding on delays in percutaneous coronary intervention for acute myocardial infarction.^24^–^26^ For sepsis care, studies have demonstrated significant delays in core treatments including time to intravenous fluids and antibiotic administration.^27^,^28^ ED crowding continues to have large implications for delays in less emergent care as well. For example, studies have found delays in community-acquired pneumonia treatment as well as increased mortality associated with increased ED crowding for these patients.^14^,^29^–^31^ Multiple studies have also demonstrated an association between increased ED volume and delays in analgesia administration; this notably includes patients with sickle cell crises.^32^–^34^ As capacity constraints continue to grow, understanding and creating better processes of care for defined patient populations may become even more essential in the ED.

One potential explanation for our findings could be that times of peak crowding occurred concurrently with times of increased resource availability. Given that most patients presenting during times of crowding arrived during the day and on a weekday, it is likely that increased hospital staffing and resource availability could contribute to expedited care during peak hours. In fact, recent studies have shown that a reduction in available physicians and nurses has been associated with increased DIT and DTN times, respectively.^35^ Reassuringly, we found that the distribution of capacity constraints for stroke patients presenting to the ED was similar to that for the general population. Further study may be warranted to better characterize how ED staffing models and time of presentation may affect delays in stroke care during times of increased ED crowding.

## LIMITATIONS

Our retrospective analysis is not without limitations. We assessed the impact of capacity constraints in a large, urban, academic center with a robust system in place for acute stroke care. This may limit generalizability, as the relationship between crowding and care delivery may vary based on practice type and resource availability. However, we believe that our findings are valuable in highlighting the potential to maintain high-quality, time-critical care delivery even in the face of major capacity challenges.

Another limitation is that our retrospective analysis may not have been powered to detect a difference in our study outcomes, despite the fact that our comprehensive stroke center sees the largest number of patients with acute stroke in our state, and we were able to capture clinical data on all acute stroke patients during the study period, we did have a relatively smaller proportion of patients presenting during times of the highest crowding. One explanation for this is that our institution is closed to outside hospital transfers during times of severe crowding, which may limit the number of patients with ischemic stroke at this time. In our large cohort, we found no trend towards significance for the study outcomes, yet it remains plausible that with larger samples, specifically for high and severe capacity constraints, there may exist a significant effect.

Additionally, with the exception of patients with LVO, our institution did not accept interfacility transfers during times of severe crowding, which narrows our study population. We also used an eight-hour window for thrombectomy consideration based on institutional protocols, although in the latter six months of the study period this expanded to 24 hours. We chose not to include the expansion because the expanded protocol was in variable implementation during that time. Our population also includes a relatively greater proportion of patients arriving via EMS as well as a relatively racially homogenous population. Findings may differ in settings with different demographics or different patterns of prehospital care.

Another limitation of our analysis is that we were unable to directly evaluate for delays to recognition of or diagnosis of stroke due to the nature of our data. However, given that our registry includes all patients with a final diagnosis of stroke, and imaging times were not different between patients presenting during times of crowding, this suggests that it is unlikely that there were substantial delays to diagnosis in these patients. Last, we could not measure the effect of prioritizing stroke care over other diseases also relying on advanced imaging to make a diagnosis. It is plausible that care for other disease states may be delayed while resources are being used for acute stroke patients.

## CONCLUSION

In our single-institution, observational study, we found that ED capacity constraints were not significantly associated with delays in acute stroke care, suggesting that robust processes of care for critically ill patients may be protective from the growing burden of ED crowding.

## Supplementary Information



## Figures and Tables

**Table 1:** Patient and clinical characteristics.

Patient and clinical characteristics	All patients n=495	Normal capacity constraints n=390 (78.8%)	High capacity constraints n=71 (14.3%)	Severe capacity constraints n=34 (6.9%)
Gender				
Female	248 (50%)	201 (51.5%)	33 (46.5%)	14 (41.2%)
Age, median (IQR)	73 (62–83)	73 (61–84)	76 (66–84)	66.5 (58–81)
Race/ethnicity				
White	358 (72.3%)	286 (73.3%)	48 (67.6%)	24 (70.6%)
Black	39 (7.9%)	26 (6.7%)	8 (11.3%)	5 (14.7%)
Asian	22 (4.4%)	17 (4.4%)	2 (2.8%)	3 (8.8%)
American Indian/Alaska Native	1 (0.2%)	0 (0%)	1 (1.4%)	0 (0%)
Hispanic	29 (5.9%)	23 (5.9%)	6 (8.5%)	0 (0%)
Unavailable	75 (15.1%)	61 (15.6%)	12 (16.9%)	2 (5.9%)
Mode of ED arrival				
Private Transport	59 (11.9%)	41 (10.5%)	10 (14.1%)	8 (23.5%)
EMS	243 (49.1%)	184 (47.2%)	39 (54.9%)	20 (58.8%)
Interfacility Transfer	193 (39.0)	165 (42.3%)	22 (40.0%)	6 (17.7%)
NIHSS on Admission#, median (IQR)	7 (2–16)	8 (3–16)	6.5 (2–12)	2.5 (1–8.5)
NIHSS > 6 on admission	304 (61.4%)	250 (64.1%)	42 (59.2%)	12 (35.3%)

*IQR*, interquartile range; *ED*, emergency department; *EMS*, emergency medical services; *NIHSS*, National Institutes of Health Stroke Scale.

**Table 2:** Study outcomes by capacity.

	All Patients n=495	Normal capacity constraints n=390 (78.8%)	High capacity constraints n=71 (14.3%)	Severe capacity constraints n=34 (6.9%)	P-value
Median DIT in minutes (IQR)	26 (17–52)	26.5 (17–54)	23 (17–37.5)	26 (17–76)	0.50
n (%)	716	222 (75%)	48 (16%)	28 (9.4%)	
Median DIT among patients receiving alteplase in minutes (IQR)	1 (14–26)	18.5 (14–26)	21.5 (12.5–32)	17 (15–20)	0.74
n (%)	82	62 (76%)	12 (15%)	8 (10%)	
Median DTN in minutes (IQR)	43 (31–59)	43 (32–60)	35 (29–47)	45 (36.5–54)	0.41
n (%)	82	62 (76%)	12 (15%)	8 (10%)	
Median DTP in minutes (IQR)	68.5 (56.5–100)	68.5 (56–100)	72 (58–95)	54	0.54
n (%)	52	46	5	1	
DIT < 25 mins among all non-transferred patients treated with alteplase					
Yes	59 (72%)	45 (73%)	7 (58%)	7 (88%)	0.45
No	23 (28%)	17 (27%)	5 (42%)	1 (13%)	
DTN < 60 mins among all patients treated with alteplase					
Yes	64 (78%)	47 (76%)	10 (83%)	7 (88%)	0.39
No	18 (22%)	15 (24%)	2 (17%)	1 (12%)	
Dysphagia Screen performed in the ED among all acute stroke patients					
Yes	864 (63%)	675 (62%)	129 (64%)	60 (64%)	0.35
No	514 (37%)	406 (38%)	74 (36%)	34 (36%)	

*DIT*, door-to-imaging time; *IQR*, interquartile range; *DTN*, door-to-needle time; *DTP*, door-to-groin puncture time; mins, minutes; *ED*, emergency department.

## References

[1] Sauser K, Levine DA, Nickles AV (2014). Hospital variation in thrombolysis times among patients with acute ischemic stroke: the contributions of door-to-imaging time and imaging-to-needle time. JAMA Neurol.

[2] Jungehulsing GJ, Rossnagel K, Nolte CH (2006). Emergency department delays in acute stroke - analysis of time between ED arrival and imaging. Eur J Neurol.

[3] Kamal N, Holodinsky JK, Stephenson C (2017). Improving door-to-needle times for acute ischemic stroke: effect of rapid patient registration, moving directly to computed tomography, and giving alteplase at the computed tomography scanner. Circ Cardiovasc Qual Outcomes.

[4] Al Kasab S, Harvey JB, Debenham E (2018). Door to needle time over telestroke: a comprehensive stroke center experience. Telemed J E Health.

[5] Kamal N, Smith EE, Jeerakathil T (2018). Thrombolysis: Improving door-to-needle times for ischemic stroke treatment: a narrative review. Int J Stroke.

[6] Ruff IM, Ali SF, Goldstein JN (2014). Improving door-to-needle times: a single center validation of the target stroke hypothesis. Stroke.

[7] Fonarow GC, Smith EE, Saver JL (2011). Improving door-to-needle times in acute ischemic stroke: the design and rationale for the American Heart Association/American Stroke Association’s Target: Stroke Initiative. Stroke.

[8] Powers WJ, Derdeyn CP, Biller J (2015). 2015 American Heart Association/American Stroke Association focused update of the 2013 Guidelines for the Early Management of Patients with Acute Ischemic Stroke Regarding Endovascular Treatment: A Guideline for Healthcare Professionals from the American Heart Association/American Stroke Association. Stroke.

[9] Jauch EC, Saver JL, Adams HP (2013). Guidelines for the Early Management of Patients with Acute Ischemic Stroke: A Guideline for Healthcare Professionals from the American Heart Association/American Stroke Association. Stroke.

[10] Trzeciak S, Rivers EP (2003). Emergency department overcrowding in the United States: an emerging threat to patient safety and public health. Emerg Med J.

[11] Derlet R, Richards J, Kravitz R (2001). Frequent overcrowding in U.S. emergency departments. Acad Emerg Med.

[12] Andrulis DP, Kellermann A, Hintz EA (1991). Emergency departments and crowding in United States teaching hospitals. Ann Emerg Med.

[13] Hoot NR, Aronsky D (2008). Systematic review of emergency department crowding: causes, effects, and solutions. Ann Emerg Med.

[14] Pines JM, Localio AR, Hollander JE (2007). The Impact of emergency department crowding measures on time to antibiotics for patients with community-acquired pneumonia. Ann Emerg Med.

[15] Mills AM, Baumann BM, Chen EH (2010). The impact of crowding on time until abdominal CT interpretation in emergency department patients with acute abdominal pain. Postgrad Med.

[16] Chatterjee P, Cucchiara BL, Lazarciuc N (2011). Emergency department crowding and time to care in patients with acute stroke. Stroke.

[17] Reznek MA, Murray E, Youngren MN (2017). Door-to-Imaging time for acute stroke patients is adversely affected by emergency department crowding. Stroke.

[18] Howard G, Schwamm LH, Donnelly JP (2018). Participation in Get With The Guidelines-Stroke and its association with quality of care for stroke. JAMA Neurol.

[19] Ormseth CH, Sheth KN, Saver JL (2017). The American Heart Association’s Get with the Guidelines (GWTG)-Stroke development and impact on stroke care. Stroke Vasc Neurol.

[20] Minnerup J, Wersching H, Unrath M (2014). Effects of emergency medical service transport on acute stroke care. Eur J Neurol.

[21] Ekundayo OJ, Saver JL, Fonarow GC (2013). Patterns of emergency medical services use and its association with timely stroke treatment: findings from Get with the Guidelines-Stroke. Circ Cardiovasc Qual Outcomes.

[22] Olascoaga Arrate A, Freijo Guerrero MM, Fernandez Maiztegi C (2019). Use of emergency medical transport and impact on time to care in patients with ischaemic stroke. Neurologia.

[23] Sauser K, Bravata DM, Hayward RA (2015). A national evaluation of door-to-imaging times among acute ischemic stroke patients within the Veterans Health Administration. J Stroke Cerebrovasc Dis.

[24] Ferrari J, Knoflach M, Seyfang L (2013). Differences in process management and in-hospital delays in treatment with IV thrombolysis. PLoS One.

[25] Harris B, Bai JC, Kulstad EB (2012). Crowding does not adversely affect time to percutaneous coronary intervention for acute myocardial infarction in a community emergency department. Ann Emerg Med.

[26] Kulstad EB, Kelley KM (2009). Overcrowding is associated with delays in percutaneous coronary intervention for acute myocardial infarction. Int J Emerg Med.

[27] Schull MJ, Vermeulen M, Slaughter G (2004). Emergency department crowding and thrombolysis delays in acute myocardial infarction. Ann Emerg Med.

[28] Gaieski DF, Agarwal AK, Mikkelsen ME (2017). The Impact of ed crowding on early interventions and mortality in patients with severe sepsis. Am J Emerg Med.

[29] Peltan ID, Bledsoe JR, Oniki TA (2019). Emergency department crowding is associated with delayed antibiotics for sepsis. Ann Emerg Med.

[30] Jo S, Kim K, Lee JH (2012). Emergency department crowding is associated with 28-day mortality in community-acquired pneumonia patients. J Infect.

[31] Fee C, Weber EJ, Bacchetti P (2011). Effect of emergency department crowding on pneumonia admission care components. Am J Manag Care.

[32] Sikka R, Mehta S, Kaucky C (2010). ED Crowding is associated with an increased time to pneumonia treatment. Am J Emerg Med.

[33] Shenoi R, Ma L, Syblik D (2011). Emergency department crowding and analgesic delay in pediatric sickle cell pain crises. Pediatr Emerg Care.

[34] Pines JM, Shofer FS, Isserman JA (2010). The effect of emergency department crowding on analgesia in patients with back pain in two hospitals. Acad Emerg Med.

[35] Sills MR, Fairclough DL, Ranade D (2011). Emergency department crowding is associated with decreased quality of analgesia delivery for children with pain related to acute, isolated, long-bone fractures. Acad Emerg Med.

[36] Tsai MT, Yen YL, Su CM (2016). The Influence of emergency department crowding on the efficiency of care for acute stroke patients. Int J Qual Health Care.

