# Supplementary material for: Impact of Emergency Department Crowding on Delays in Acute Stroke Care
Source: West J Emerg Med. 2020 Jul 8;21(4):892–9. doi: 10.5811/westjem.2020.5.45873 (PMC7390586; doi:10.5811/westjem.2020.5.45873)
Supplement: Supplementary file 1 [file wjem-21-892-s001.docx]

**Supplementary Table.** Factors independently associated with time-based stroke metrics in regression models.

|  | Time-based Stroke Metrics | | | | | |
| --- | --- | --- | --- | --- | --- | --- |
|  | Door-to-Imaging Model | | Door-to-Needle Model | | Door-to-Puncture Model | |
|  | change in time (minutes) per unit change in the variable* | P value | change in time (minutes) per unit change in the variable^#^ | P value | change in time (minutes) per unit change in the variable^+^ | P-value |
| High capacity | -30.3 | 0.17 | -6.56 | 0.45 | 5.70 | 0.69 |
| Severe capacity | 21.8 | 0.42 | -6.62 | 0.52 | -43.6 | 0.16 |
| Age per year | 0.09 | 0.88 | -0.10 | 0.65 | 0.05 | 0.87 |
| Female | 16.4 | 0.32 | 10.1 | 0.15 | -6.7 | 0.87 |
| Race W vs NW | 5.78 | 0.87 | -7.01 | 0.58 | 7.3 | 0.95 |
| Hispanic | -38.5 | 0.06 | -6.94 | 0.52 | -29.4 | 0.78 |
| EMS | -3.30 | 0.01 | -0.89 | 0.07 | 1.35 | 0.12 |

*Includes 286 patients with complete data and non-transfers, potentially eligible for alteplase or endovascular therapy.

^#^Includes 82 patients who were not transferred and were treated with alteplase within 4.5 hours of presentation.

^+^Includes 52 patients who received endovascular therapy.

*Race W vs NW,* Race white versus non-white; *EMS,* emergency medical services.
